# Vitreous inflammatory and angiogenic factors on patients with proliferative diabetic retinopathy or diabetic macular edema: the role of Lipocalin2

**DOI:** 10.1186/s12886-022-02733-z

**Published:** 2022-12-19

**Authors:** Georgios Batsos, Eleni Christodoulou, Evita Evangelia Christou, Petros Galanis, Andreas Katsanos, Loren Limberis, Maria Stefaniotou

**Affiliations:** 1 Faculty of Medicine, Department of Ophthalmology, University of Ioannina, 45110 Ioannina, Greece; 2 Clinical Epidemiology Laboratory, National and Kapodistrian University of Athens, Athens, Greece; 3 Department of Engineering, East Carolina University, Greenville, NC USA

**Keywords:** Lipocalin-2, Diabetic macula edema, Inflammation, Angiogenic factors, Vitreous

## Abstract

**Purpose:**

Quantitative analysis of vitreous inflammatory and angiogenic factors from patients with proliferative diabetic retinopathy (PDR) or diabetic macular edema (DME).

**Materials and methods:**

Collection of undiluted vitreous samples from 20 diabetic patients: 13 with proliferative diabetic retinopathy (PDR) and 7 with diabetic macular edema (DME). DME patients had suboptimal response to anti-VEGF treatment. Samples from 11 control patients, with vitreomacular interface pathology such as idiopathic epiretinal membrane (iERM) (*n* = 4), vitreomacular traction syndrome (VMT) (*n* = 3) and full thickness macular hole (FTMH) (*n* = 3), were also collected. The levels of IL1b, IL6, IL8, IL27, TNFα, ICAM-1, VCAM, MCP-1, VEGFA and LCN2 were measured using cytometry flow analysis. Median values were compared with Mann–Whitney test since the distributions were skewed. Statistical analysis was performed with the Statistical Package for Social Sciences software (IBM Corp. Released 2012. IBM SPSS Statistics for Windows, Version 21.0. Armonk, NY: IBM Corp.).

**Results:**

The median concentration of LCN2, IL6, IL8, IL1b, IL27, ICAM, VCAM-1, MCP-1, TNFa and VEGFA was higher in PDR patients than in controls. Similarly, the median concentration of LCN2, IL6, IL8, IL27, ICAM, VCAM-1, TNFa and VEGFA was higher in DME patients than in controls. In particular, median LCN2 concentration in diabetic patients was 5,711 pg/ml (interquartile range [IR] = 2,534), while in controls was 2,586 pg/ml (IR = 2,345). Moreover, median LCN2 was 6,534 pg/ml in the DME group (IR = 6,850) and 4,785 pg/ml in the PDR group (IR = 2,608), (*p* = 0.025).

**Conclusion:**

Various inflammatory and angiogenic factors are involved in the pathophysiology of PDR and DME. Elevated vitreous levels of LCN2 in PDR and especially in DME patients reveal a potential pathogenic association. More extended studies could verify LCN2 as an alternative therapeutic target.

## Introduction

Diabetes mellitus (DM) is a metabolic systemic disease affecting 422 million people worldwide [1]. Proliferative diabetic retinopathy (PDR) and diabetic macular edema (DME) are serious, vision threatening complications of DM [2]. There are approximately 17 million people with PDR and 28 million people with DME worldwide [3]. In the next years, the prevalence of these complications is expected to increase [4].

Both PDR and DME are considered consequences from diabetic retinopathy (DR), which is the result from a consecutive process between vascular alterations and inflammation [5–9].

Anti-vascular endothelial growth factor (anti-VEGF) agents remain the gold standard treatment for DME patients [10]. Yet, 30% of DME patients show suboptimal response with anti-VEGF therapy [11]. In clinical practice, the inflammatory component of DME can be addressed with steroids, such as dexamethasone implants [12–14]. Regarding PDR patients, recent studies have presented very favorable results with anti-VEGF agents (Protocol S, Clarity Study) [15, 16]. Yet, research is focusing on various factors as potential therapeutic targets for DME and PDR [7, 8, 17, 18].

Lipocalin-2 (LCN2), also described as neutrophil gelatinase-associated lipocalin (NGAL) [19], is a glycoprotein with a pleotropic action in different processes such as metabolism and inflammation [20]. It is also regarded as biomarker in various diseases, such as multiple sclerosis, acute kidney injury, lupus nephritis, cardiovascular disease and others [20]. Elevated serum LCN2 levels have been identified on patients with type 2 DM [21]. Serum LCN2 levels are also positively correlated with DR in these patients [22]. The role of LCN2 in neurological complications of diabetes has also been studied [23]. We have previously found a significant correlation between vitreous LCN2 and proliferative vitreoretinopathy (PVR) grade [24]. Recent data have shown upregulated vitreous NGAL in ocular sarcoidosis [25]. A significant increase of vitreous LCN2 and a correlation with VEGF has also been found in PDR patients [26]. Yet, the role of LCN2 in DME has not been studied thus far. The aim of our study was to investigate a potential association of LCN2 with DME or PDR in conjunction with other inflammatory and angiogenic factors.

## Materials and methods

### Study design

This study was conducted at the University Hospital of Ioannina, Greece, between March and September 2019. Approval was received from the hospital’s ethics committee “Scientific Board” (March 2019). All patients were recruited and examined at the University Ophthalmology Clinic. Written informed consent was obtained from each patient during the recruitment period and before the operation. The study is adherent to the tenets of the Declaration of Helsinki.

In this study, we have collected vitreous samples from 20 diabetic and 10 control patients. From diabetic patients, 13 had PDR and 7 DME. The control group included 4 patients with idiopathic epiretinal membrane (iERM), 3 with vitreomacular traction syndrome (VMT) and 3 with full-thickness macular hole (FTMH). In all patients, complete ophthalmic examination, including optical coherence tomography (OCT) and fluorescein angiography (FA) was performed. All DME patients had previously received anti-VEGF treatment (Ranibizumab or Aflibercept) with a suboptimal response. These eyes had OCT-central subfield thickness (OCT-CST) of 250 μm for a period of 24 weeks and received at least 4 intravitreal anti-VEGF injections [27]. We did not notice anatomical improvement after switching between Ranibizumab and Aflibercept. No significant vitreoretinal interface pathology (such as ERM) was confirmed by OCT in DME cases. Fundoscopy revealed no tractional component in all diabetic patients, while in cases of vitreous hemorrhage (VH) coexistence, ultrasonography (US) was performed in order to exclude any tractional retinal detachment. Apart from panretinal photocoagulation (PRP), no other treatment had been applied to PDR patients. Diabetic patients were divided in two groups (PDR and DME) because they have received different treatment (intravitreal injections or PRP). DME patients did not had proliferative disease. The exclusion criteria included ocular trauma history, prior ophthalmic surgery other than phacoemulsification, ocular or systemic inflammation and malignancy.

Each operation was performed by the same vitreoretinal surgeon and included standard 25G pars plana vitrectomy with Alcon Constellation system. Each sample (0.5 ml of core vitreous) was collected before opening the infusion cannula and stored at -80 °C. Then, all samples were analyzed with cytometry flow for the quantification of IL1b, IL6, IL8, IL27, TNFα, ICAM-1, VCAM, MCP-1, VEGFA and LCN2 (NGAL). According to literature, these factors are implicated in the pathophysiology of PDR or DME development [26, 28–30], thus a potential correlation with LCN2 concentration could highlight an inflammatory or angiogenic pathway association. In DME patients, vitrectomy was performed in order to achieve better oxygenation and removal of the angiogenic and inflammatory factors from the macular area [31]. Moreover, in order to achieve complete vitreous cortex removal [31] and avoid postoperative ERM development [32] peeling of the internal limiting membrane (ILM) using brilliant blue dye was performed. No postoperative complications were noticed in all cases.

### Cytometry flow analysis

Vitreous samples were tested with the AimPlex multiplex assay in a FACSCalibur (Becton–Dickinson) cytometer. Results were analyzed with the CellQuest software (Becton–Dickinson). The technology of AimPlex multiple analysis works by using multiple populations of beads that differentiate in size and level of fluorescence intensity. In this way, multiple molecules can be measured during the same reaction. Cytometric bead array is based on the same principle as sandwich ELISA. Every bead population is conjugated with a specific antibody, which can bind with the target analyte (cytokine).

**Fig. 1:**
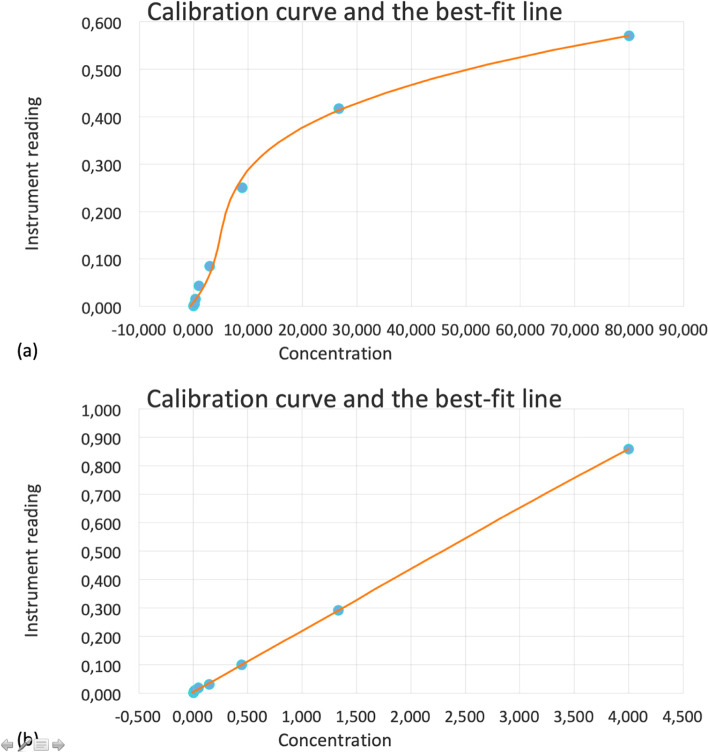
Standard curves of LCN2 (**a**) and VEGFA (**b**). The horizontal axis corresponds to the concentration in pg/ml and the vertical axis the mean fluorescence intensity (MFI)

**Fig. 2:**
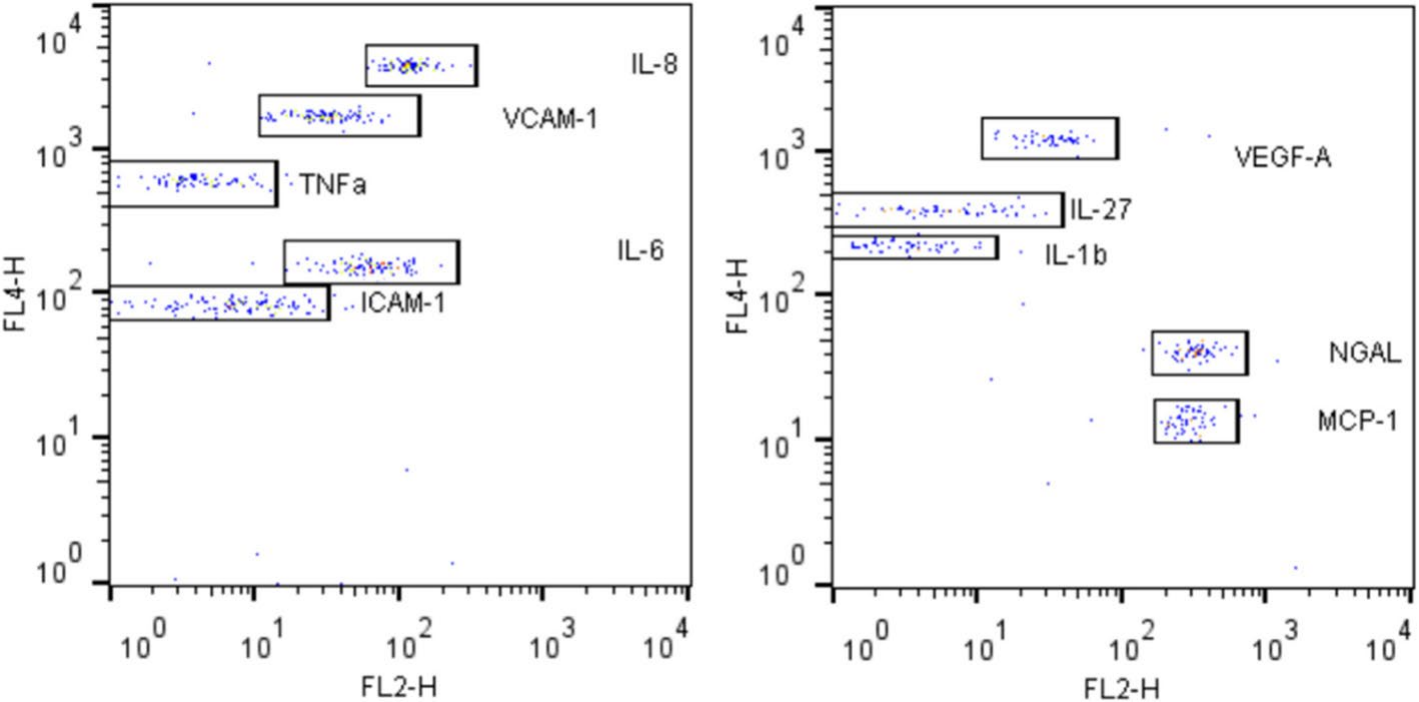
Dot-plot diagrams of the measured factors from a DME case. The horizontal axis (FL2) represents the fluorescent intensity related to the concentration of each measured factor (IL1b, IL6, IL8, I-CAM, V-CAM, MCP-1, TNFa, VEGF, NGAL) framed in a box. The vertical axis (FL4) represents the fluorescent intensity serving for distinguishing each factor, by allocating them on different sites in the plot

Concentrations were obtained from the measured fluorescent intensity after comparison with the fluorescent signal of a standard curve. The standard curve corresponds to 8 measurements from a solution with a known analyte concentration (cytokine) (Fig. 1). Results were expressed as picograms per milliliter (pg/ml). The first step of the test included the incubation of the antibody bearing beads with the antigen for 60 min, which was followed by the biotinylated antibody incubation for 30 min. The last step included the streptavidin phycoerythrin incubation for 20 min. Figure 2 illustrates the dot-plot diagrams of the measured factors, from a DME case.

### Statistical analysis

Continuous variables are presented as mean, standard deviation, median, interquartile range, minimum value and maximum value, while categorical variables are presented as percentages (frequencies). Continuous variables did not follow the normal distribution and thus nonparametric methods were used. For continuous variables, differences between the two groups were evaluated with Mann–Whitney U test. Variability between the DR and the control group in terms of age and gender was assessed with independent-samples t-test and chi-square test respectively. A two tailed p-value of less than 0.05 was considered statistically significant. Statistical analysis was performed with the Statistical Package for Social Sciences software (IBM Corp. Released 2012. IBM SPSS Statistics for Windows, Version 21.0. Armonk, NY: IBM Corp.).

## Results

There were no differences in age and gender between diabetic patients and controls. In particular, mean age in the diabetic patients group was 67.6 years (standard deviation [SD] = 11.9, minimum value = 33, maximum value = 87) while in the control group was 68.4 years (SD = 10.5, minimum value = 51, maximum value = 84) (t = 0.2, *p* = 0.9). Mean age for the DME group was 70.6 years (SD = 8.9, minimum value = 57, maximum value = 87) while for the PDR group was 65.1 (SD = 13.7, minimum value = 33, maximum value = 86). In diabetic patients group 55% (*n* = 11) were males, while 30% (*n* = 3) of controls were males (x^2^ = 1.7, *p* = 0.26). Mean HbA1c for DME was 7.4% (SD = 2.1%, median = 6.8%, minimum value = 5.1%, maximum value = 12%), while for PDR was 9.5% (SD = 1.6%, median = 10%, minimum value = 7%, maximum value = 12%), (t = 2.5, *p* = 0.02).

**Fig. 3:**
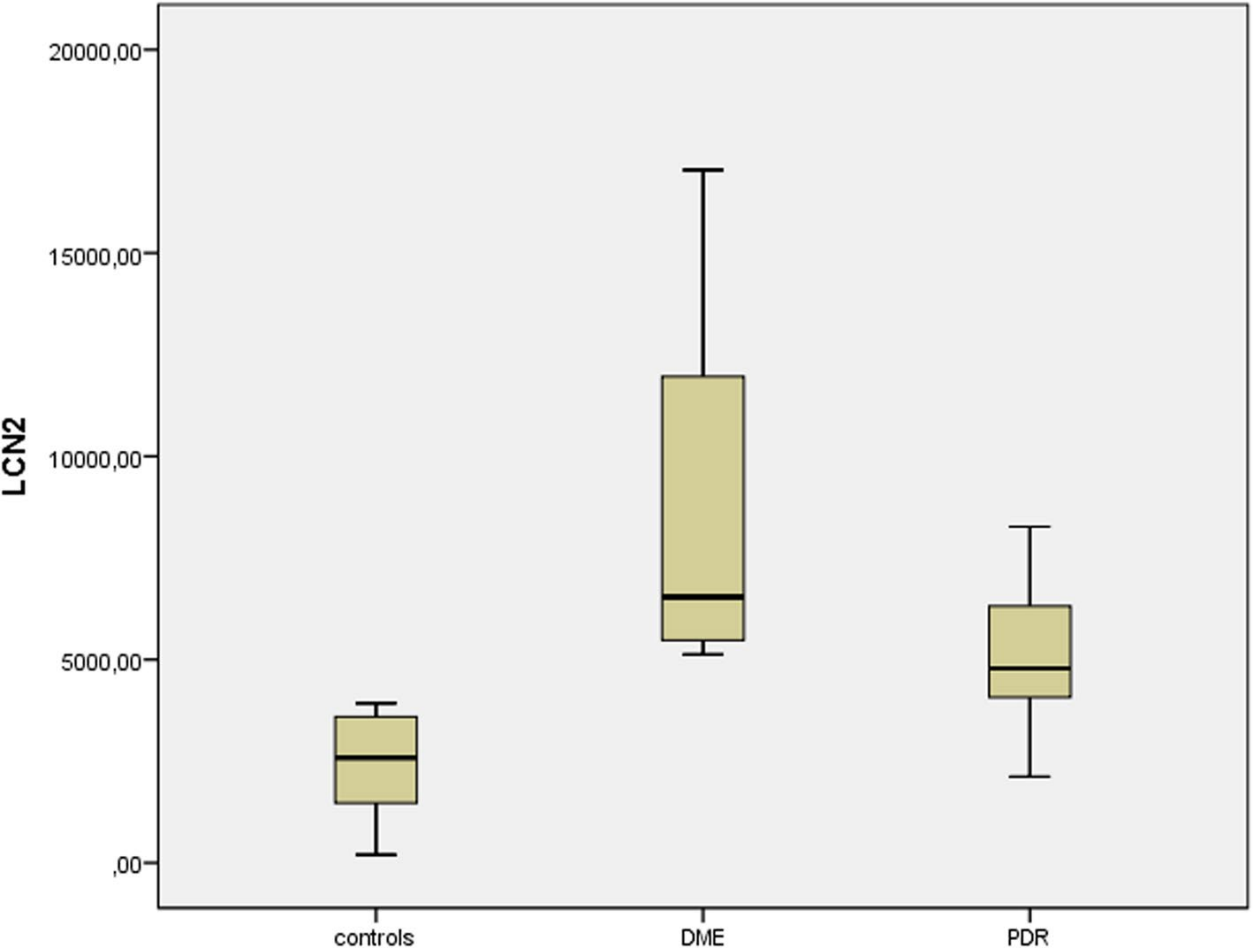
LCN2 levels of the controls, DME and PDR patients

Relations between measured factors and patients are shown in Table 1. The median concentration of LCN2, IL6, IL8, IL1b, IL27, ICAM, VCAM-1, MCP-1, TNFa and VEGFA was higher in PDR patients than in controls. Also, the median concentration of LCN2, IL6, IL8, IL27, ICAM, VCAM-1, TNFa and VEGFA was higher in DME patients than in controls. In particular, median LCN2 concentration in in diabetic patients was 5,711 pg/ml (interquartile range [IR] = 2,534), while in the controls was 2,586 pg/ml (IR = 2,345). Also, median LCN2 was 6,534 pg/ml in the DME group (IR = 6,850) and 4,785 pg/ml in the PDR group (IR = 2,608). LCN2 levels of the patients and the controls are shown in Fig. 3. There were no outliers.

**Table 1:** Relations between measured factors and patients

	Mean	Standard deviation	Median	Minimum value	Maximum Value	Interquartile range	*P*-value^a^
LCN2							
Controls	2,351	1,291	2,586	200	3,921	2,345	
Diabetic Patients	6,655	3,509	5,711	2,116	17,042	2,534	< 0.001^b^
DME	8,581	4,246	6,534	5,126	17,042	6,850	< 0.001^c^
PDR	5,078	1,703	4,785	2,116	6,149	2,608	0.001^d^
IL6							
Controls	57.6	80.3	13.5	1.28	224.1	111.1	
DME	143.8	119.2	106.6	28.4	370.4	172.3	0.03^c^
PDR	144.2	75.8	138.5	31.7	323.5	82.1	0.016^d^
IL8							
Controls	85.5	113.5	63.5	3.9	390.6	83.5	
DME	349.9	292.7	271.2	70.4	1,027	328.5	0.002^c^
PDR	1,201	1,327	658	56.2	4,750	977	< 0.001^d^
IL1b							
Controls	5.1	1.1	5.1	3.3	6.9	1.8	
DME	8.3	6.7	5.5	3.8	25.2	4.4	0.182^c^
PDR	8.9	2.8	8.3	6.9	16.9	1.7	< 0.001^d^
IL27							
Controls	23.6	12.1	18.7	10.7	53.5	12	
DME	42.5	25.6	30.3	18.1	100.4	31.1	0.028^c^
PDR	52.9	18.3	50.7	24.6	96.7	6	0.001^d^
ICAM							
Controls	98.8	83.8	64	50	264	64.2	
DME	343.8	298.5	260.6	60	935.9	449.3	0.006^c^
PDR	358.1	258.1	267.5	90	830	353	0.001^d^
VCAM-1							
Controls	395	212.4	333.3	170.4	897	145.7	
DME	1,087	625.3	862	342.4	2,200	988	0.002^c^
PDR	887	322.3	776	516.4	1.404	678	0.001^d^
MCP-1							
Controls	505.4	357.2	414.6	11	1050.2	583.3	
DME	897.7	427.1	775.4	343.4	1,751	587.4	0.065^c^
PDR	1,076	316	1,150	595.3	1,480	653	0.002^d^
TNFa							
Controls	11.9	3.2	10.7	9.2	19.8	3.2	
DME	17	5.9	14.3	10.6	29.4	7.9	0.01^c^
PDR	21.2	6	20	12.7	35.3	5.8	< 0.001^d^
VEGFA							
Controls	34.3	13	32.7	11	60.3	13	
DME	541.2	494.5	341	15.6	1,273	990	0.008^c^
PDR	599.5	642.7	388	34.2	2,275	770	< 0.001^d^

^a^Mann-Whitney test

^b^controls vs. Diabetic patients

^c^controls vs. DME

^d^controls vs. PDR

Median LCN2 was higher in DME group than PDR group (*p* = 0.025), while median IL8 was higher in PDR group than DME group (*p *= 0.02). Medians’ LCN2 difference between DME and controls (3,948) was almost twice higher than medians’ LCN2 difference between PDR and controls (2,199). Also, medians’ IL8 difference between PDR and controls (594.5) was almost three times higher than medians’ IL8 difference between DME and controls (207.7).

## Discussion

In this study, we found elevated LCN2 levels in the vitreous of diabetic patients. Median LCN2 concentration was higher in PDR and markedly higher in DME group as compared to controls. To our knowledge, this observation has not been reported to the literature so far.

Numerous angiogenic and inflammatory factors are implicated in the pathogenesis of DR, and therefore in DME and PDR [5, 6, 8]. The NF-κβ pathway is a key element in the development of vascular complications caused in DM and DR and it is related to the expression of IL1b, IL6, IL8, TNFa, I-CAM and MCP-1 [33]. In a recent study, it has been proposed that intravitreal LCN2 can suppress ocular inflammation (in rat models) by inhibiting the activation of NF-κβ pathway [34]. The anti-inflammatory role of LCN2 in macrophages and NF-κβ pathway has also been reported before [35].

The vascular alterations in DR are accompanied by blood retinal barrier (BRB) breakdown leading to DME development [36]. The BRB breakdown can develop, due to the junctional protein damage and the vascular endothelial cell dysfunction [37]. The role of LCN2 in vascular endothelial cell function has been studied in the cerebrovascular system [38]. It has been proposed that LCN2 might reduce the damage to endothelial junctional proteins (ZO-1, VE-cadherin) after ischemic brain stroke, acting as an endogenous ‘help me signal’ and thus maintaining the blood brain barrier (BBB) integrity [38]. On the other hand, LCN2 can also promote angiogenesis [39, 40]. For this reason, the specific role of LCN2 in both PDR and DME needs to be elucidated with further studies; in fact, its role in modulating pro- and anti-inflammatory responses is still under research [41].

Matrix Metalloproteinase-9 (MMP-9) has a key role in the pathogenesis of DR and progression to PDR [42–46]. MMP-9 also correlates both with the DME development [47] and the structural damage caused by chronic DME [47]. At the same time, LCN2 modulates the activity of MMP-9 [48–51], highlighting the rationale for further investigation in this field.

We have also found a significant elevation of IL27 in DME and PDR patients as compared to controls. Elevated IL27 levels have been previously measured in the aqueous humor of patients with diabetic retinopathy [29]. The anti-inflammatory role of IL27 in ocular inflammation has also been described [52]. VEGFA is a potent angiogenic factor, which can also act as a chemoattractant to macrophages and granulocytes, or can induce vasodilation [53]. Q Zhang et al. reported that IL27 can suppress the VEGFA production in macrophages on patients with diabetic retinopathy [54].

To date, there is limited evidence concerning the role of LCN2 in DM. Conceivably, this information is be considered relevant for clinical practice, as this glycoprotein could serve as a predicting factor for the prognostication in diabetic patients and as a potential therapeutic target. The aforementioned parameters along with our observation concerning increased levels of LCN2 in DME may add strength to our study. In any case, our study contributes to a little-studied issue that warrants further investigation.

Undoubtedly, the results of the present study should be interpreted with certain limitations. Firstly, our study sample consists of a small number of recruited patients. However, this sample size enabled significant differences. The patients with DME without concurrent vitreoretinal pathology (such as ERM) and indication for vitrectomy are rare cases. Thus, even with few recruited patients, this study provides very useful information for clinical practice. Undoubtedly, the small sample does not allow us to conclude if the LCN2 concentration differences can be attributed to the different treatment strategies in each group (either PRP in PDR or anti-VEGF in DME). Another limitation concerns the levels of VEGFA at the patients with DME. All patients with DME had received anti-VEGF treatment before. This is because one of the study aims was to investigate alternative therapeutic targets in cases that are refractory to anti-VEGF treatment. Lastly, caution is needed in the interpretation of results concerning the quantification of VEGF (with antibody based assays) in the presence of antibody-based anti-VEGF agents, such as Ranibizumab or Aflibercept [55, 56].

In conclusion, we have found elevated vitreous LCN2 levels in patients with PDR and in patients with DME refractory to anti-VEGF treatment. These findings are accentuating the role of LCN2 in the pathogenesis of PDR and DME, adding further information to previous studies. Larger longitudinal studies are needed, in order to determine the significance of LCN2 as biomarker or therapeutic target.

## Data Availability

The datasets used and analyzed in the current study are available from the corresponding author on reasonable request.
